# Health care across the first year postpartum and experiences of women with anxiety/depressive symptoms: A longitudinal cohort of first-time mothers in Ireland (MAMMI)

**DOI:** 10.1007/s00737-025-01670-2

**Published:** 2026-02-05

**Authors:** Susan Hannon, Deirdre Gartland, Agnes Higgins, Stephanie J. Brown, Deirdre Daly

**Affiliations:** 1 School of Nursing and Midwifery, Trinity College Dublin, 24 D’Olier Street, Dublin, Trinity, DO2 T283 Ireland; 2 Trinity Centre for Maternity Care Research (TCMCR), School of Nursing and Midwifery, Trinity College Dublin, No. 2 Clare Street, 2, Dublin 2, Ireland; 3 Intergenerational Health, Murdoch Children’s Research Institute, University of Melbourne, Melbourne (Vic), Australia; 4 Department of General Practice, University of Melbourne, Melbourne (Vic), Australia; 5 Department of Paediatrics, University of Melbourne, Melbourne (Vic), Australia

**Keywords:** Help-seeking, Perinatal mental-health, Maternal wellbeing, Healthcare systems

## Abstract

**Background:**

Women’s increased contact with healthcare professionals during pregnancy and the early postpartum make it an opportune time to identify any physical or mental health problems that emerge during this period, yet mental health problems are under-identified in clinical settings. This study uses quantitative and qualitative survey data from a large prospective study of first-time mothers in Ireland to investigate contacts with healthcare professionals, enquiry about mental health, and women’s satisfaction with the support they received.

**Methods:**

Quantitative and qualitative data were collected during pregnancy and at 3, 6, 9 and 12 months postpartum. The sample comprised 2572 mothers reporting on postpartum anxiety/depressive symptoms using the Depression, Anxiety and Stress Scale.

**Results:**

Approximately 50–70% of women reported that a healthcare professional asked about their mental health at three months postpartum. Contact with healthcare professionals and enquiries about maternal health decreased significantly over the course of the first 12 months. Women felt that postpartum care was concluded too soon, and that appointments were often hurried and infant-focused. Women reporting anxiety/depressive symptoms had more frequent appointments for their own and their baby’s health than women who did not report symptoms. Women with symptoms were more likely to indicate they felt uncomfortable addressing sensitive topics and worried about the consequences of disclosure. Many women described positive and supportive interactions with professionals; others reported that their disclosures were dismissed, while some feared that disclosures may lead to judgment, labelling or being recommended psychotropic medication as a solution.

**Conclusions:**

The findings support the need for an expansion of the postpartum care currently provided in Ireland. Greater support for health professionals regarding asking and responding to women’s needs across the postpartum period is required to address mothers’ hesitancy to share concerns and to broaden access and pathways of care.

**Supplementary Information:**

The online version contains supplementary material available at 10.1007/s00737-025-01670-2.

## Highlights

Although provided as a standard and free part of Irish postpartum maternity care, this research found that a significant percentage of women did not receive or attend a postpartum health check with a GP for themselves (n=583, 23.9%) or their baby (n= 311, 12.95%) in the first three months postpartum.

This research found a correlational association between reporting anxiety/depressive symptoms and the number of visits to a Healthcare Professional (HCP). Women who reported anxiety/depressive symptoms attended more GP visits for their own health at six (OR=3.6, 95%CI 2.4 -5.4), nine (OR=4.6, 95%CI2.7 -7.5), and 12 (OR=3.3, 95%CI 1.9 -5.5) months.

Medical and child development appointments in the postpartum period are opportunities for healthcare professionals to enquire about mothers’ physical and mental wellbeing and provide appropriate care or referral. However, only 51.7% of women reported that their GP enquired if they were feeling depressed or low in the postpartum period.

Women who reported anxiety/depressive symptoms were less likely to perceive their HCPs as easy to talk to about concerns, and more likely to endorse statements indicating fears of being judged or facing undesirable repercussions following disclosure, than women who did not report mental health symptoms.

Women reported that postnatal care is predominately baby-focused, and advocate for additional postnatal care pathways that extend beyond three months postpartum and that provide an opportunity for greater emotional support for mothers. Women indicated that having access to a healthcare professional, dedicated maternal/baby healthcare spaces, more frequent appointments and/or call lines would be beneficial to women in the postpartum period.

**Supplementary Information:**

The online version contains supplementary material available at 10.1007/s00737-025-01670-2.

## Introduction

While many women experience the transition from pregnancy to motherhood in good health; pregnancy and the postpartum period are associated with, and represent a risk for, experiencing a range of physical (Daly et al., 2018; Gartland et al., 2016; Wuytack et al., 2018) and mental health problems (Falah-Hassani et al., 2017; Kuipers et al., 2021). Globally, mental health problems such as anxiety and depression are the most common health concerns occurring in the perinatal period (Howard & Khalifeh, 2020). Prevalence estimates of depression in the antenatal and postpartum periods are between 17.2% and 13.1% (Underwood et al., 2016), while estimates for anxiety disorders range from 4.5% to 15% (Paschetta et al., 2014). The implications of maternal mental health problems for women’s wellbeing (Li et al., 2022), their relationships (Yeaton-Massey & Herrero, 2019), and child development (Netsi et al., 2018; O’Connor et al., 2016; von Hinke et al., 2022; Zhang et al., 2023) have been well recognised. Additionally, the interplay between physical illness and mental illness is increasingly documented; physical illness is a potential risk factor for mental ill health (Jang, 2024; Ohrnberger et al., 2017), and should therefore prompt healthcare professionals to ask women about their mental health experiences of physical health issues.

In the perinatal period, women may have contact with a range of healthcare professionals including midwives, nurses, public health nurses, physiotherapists, general practitioners, obstetricians, psychiatrists, psychologists, and social workers. Women’s increased contact with healthcare professionals during pregnancy and the early postpartum period make it an opportune time to identify any health problems and to connect women with suitable treatments, services or interventions. However, despite the prevalence and impact of perinatal mental health problems, the literature also suggests that they are under-identified by healthcare professionals. For example, research has found that as few as 50% (Chaudron et al., 2004) and 30% (Cox et al., 2016) of women experiencing depression are identified in clinical perinatal settings. Under-identified perinatal mental health problems result in untreated and inadequately treated mental health problems and fewer instances of recovery (Cox et al., 2016). Without identification and corresponding health care response, mental health issues are unlikely to resolve and may increase in severity, with on-going implications for the mother, child and family (Falah-Hassani et al., 2017), and result in considerable social and economic burden (Bauer et al., 2024; Margiotta et al., 2022; Moran et al., 2020).

Under-identification is perhaps compounded by women’s reluctance to disclose mental health issues. Women are less likely to seek out support or disclose issues with mental health in the perinatal period (Sorsa et al., 2021) due to stigma, concerns of being labelled or defined by their mental health, or the consequences it may have for their family (Dolman et al., 2013; Nagle & Farrelly, 2018). Women report being worried that they will be viewed as weak, inadequate, or a potential threat to the safety of their child or children (Bilszta et al., 2011; Mule et al., 2022) and that disclosure may instigate child protection procedures (Goldberg & Frost, 2025; Jones, 2022). These factors can encourage women to remain silent about their distress.

The Maternity and Infant Care Scheme provides free pregnancy and postpartum care to all women ordinarily resident and giving birth in the Republic of Ireland. The scheme offers combined care and services that are shared by a woman’s general practitioner (GP) and midwife or obstetrician in a maternity hospital. During pregnancy, women will receive an initial examination with their GP followed by additional antenatal appointments, which alternate between the GP and maternity hospital’s midwife or obstetrician. In the postpartum period, the scheme provides for one GP visit two weeks after birth for the baby’s health, and a GP visit for both the baby’s and mother’s health six weeks after birth (Department of Health, 2016). In addition, women receive a visit from a public health nurse (PHN) within 72 hours of discharge from the maternity hospital to check on both mother and infant (Giltenane et al., 2022). This PHN visit is deemed a mandatory health service provision; however, missed care is not uncommon due to disparity between staff and task-load volume (Phelan et al., 2018). Typically, a PHN will also carry out, non-mandatory, child developmental checks at 3 months and 9–11 months after birth (Department of Health, 2016; Executive, 2023).

At present, there is no national policy for mental health screening among pregnant and postpartum women in Ireland, despite recommendations from both consumer and professional bodies (Health Services Executive, 2017). Although Irish maternity hospitals may integrate mental health screening as part of antenatal care (Department of Health, 2016), procedures vary from hospital to hospital and it is not known how widely it is implemented. Published Irish findings are not encouraging. For example, studies show that healthcare professionals engage in selective screening in the antenatal period and many women are not asked about their mental health (Carroll et al., 2018; Higgins et al., 2018; Noonan, Jomeen, et al., 2018). This represents a missed opportunity to identify difficulties and provide appropriate care or referral. Gaps have also been identified where mental health problems identified in the antenatal period are not communicated to those caring for women in the postpartum period (Higgins et al., 2016). These findings have also been reported for the postpartum period (Association for Improvements in the Maternity Services Ireland (AIMSI), 2010). This is especially concerning as recently published Irish perinatal mental health data found higher rates of clinically significant symptoms of depression and stress at three, six, nine and twelve months postpartum than were recorded during pregnancy (Hannon, Gartland, et al., 2022).

The current study aims to establish the state and extent of postpartum healthcare and support received by first-time mothers giving birth in Ireland throughout the first postpartum year. By drawing on quantitative and qualitative data from the MAMMI (Maternal health and Maternal Morbidity in Ireland) study, Ireland’s first national, multicentre prospective cohort study it investigates: (i) the frequency of contact with Midwives, PHNs and GPs/doctors across the first year postpartum and enquiry about mental health; (ii) women’s satisfaction with postpartum healthcare and support; (iii) the frequency of contact by maternal anxiety and/or depressive symptoms; (iv) barriers and facilitators to postpartum healthcare and support in relation to mental health/sensitive issues.

These research aims are underpinned by the knowledge that timely identification of mental health problems and receiving adequate professional support is essential to both maternal and infant wellbeing. Ireland’s first National Maternity Strategy (Department of Health, 2016) represents a governmental commitment to maternal health in Ireland, however limited data exists in an Irish context to establish the frequency of women’s contact with healthcare professionals postpartum, whether enquiries about mental health are common practice among healthcare professionals during this period, and women’s satisfaction with interactions and postpartum supports, and factors that influence women’s willingness to engage in help-seeking behaviour in the form of disclosure to healthcare professionals. Documenting, and understanding these issues as they exist contextually to Ireland, is therefore of substantial importance. The findings, in terms of the strengths and weaknesses of the current postpartum service provision, may inform and guide the development of the next iteration of the National Maternity Strategy, and contribute to better policy and service provision that is equipped to provide responsive and accessible postpartum care to improve mothers’ and infants’ health.

## Methods

### Design

This study uses data from the MAMMI study. The MAMMI study is a longitudinal cohort study with antenatal and postnatal self-completion surveys, hospital maternity records (from consenting women), and qualitative investigations into specific morbidities or maternal health issues. For example, urinary incontinence, pelvic girdle pain, sexual health problems, maternal health research and positive postpartum wellbeing (Daly et al., 2019; Daly et al., 2021; Hannon, Newnham, et al., 2022; O’Malley et al., 2019; O’Malley et al., 2022; O’Malley et al., 2018) (For detailed information, see https://www.tcd.ie/mammi/).

### Recruitment

At their first antenatal appointment in three maternity hospitals, midwives offered eligible women the MAMMI study information pack. Eligible women were nulliparous (women who have not had a previous live birth), 18 years of age or older, and had sufficient literacy in English to complete the surveys. Approximately 8,243 women were offered the study information between January 31 st 2012 and March 31 st 2017 and 3,131 participants completed the enrolment survey (response rate: 37.98%). Retention to the study was 87% at 3 months postpartum. Full recruitment and retention details have been published elsewhere (Hannon, Gartland, et al., 2022). Ethics approval was granted by the university’s research ethics committee and each of the three hospital’s ethics committees. Written informed consent was obtained from all participants. The cohort enrolled into the study is broadly representative of the population giving birth in Ireland at the time of recruitment with regards to maternal region of birth and employment status, however there are proportionately more participants in the 30–34 year old age-range in comparison to national figures for first-time mothers (Central Statistics Office, 2016). Additionally, two-thirds of women reported having attained a third-level education or equivalent, whereas approximately half of women aged 25 to 44 in the general population had attained the same levels of education (Central Statistics Office, 2017).

### Measures

 Mental health symptoms were assessed with the short-form Depression, Anxiety and Stress Scale (DASS-21) (Lovibond, 1995), which was completed in pregnancy, three, six, nine and twelve months postpartum. The DASS-21 offers a means of measuring the symptoms of depression, anxiety, stress, and comorbid anxiety and depression; however, it should be noted that it does not indicate a diagnosis. The three subscales of depressive, anxiety and stress symptoms are made up of 7 items each, with each item rated from 0 “*Did not apply to me at all*” to 3 “*Applied to me very much or most of the time*”. This paper employed the anxiety and depression subscales. Good reliability and discriminant validity have been reported (Brown et al., 1997; Crawford & Henry, 2003; Henry & Crawford, 2005), and it is suitable for use among pregnant (Xavier et al., 2016) and postpartum (Miller et al., 2006) populations.

As per the scoring instructions, scale items were summed to create a total scale score. This score was dichotomised into *none* or *low* symptoms versus *moderate* to *severe* symptoms using the recommended cut-off scores (Lovibond & Lovibond 1995, Lovibond 1995), (e.g. Depression: ≥7. Anxiety: ≥6).) (Henry & Crawford, 2005; Lovibond, 1995). Scores above these values are indicative of clinically significant levels of psychological distress. Women were then identified as experiencing ‘neither’ or as experiencing ‘anxiety and/or depression’ at each time point. This will be referred to as anxiety/depressive symptoms from this point on. This approach has been applied previously with postpartum populations (Hannon, Gartland, et al., 2022; Hannon et al., 2023).

#### Visits with health services in pregnancy and postpartum

At each time point, the survey asked women how many times in the past three months they had been visited by or had visited a local doctor/GP, midwife and or PHN. Visits to the doctor/GP comprised two questions that specified visits regarding their own health, and visits about their baby’s health. Response options were ‘Never’, ‘Once’, ‘Twice’, ‘Three times’, ‘Four times’, ‘5–6 times’ or ‘7 or more times’ and were combined into three categories: ‘Never’, ‘1–2 times’ and ‘3 or more times’.

For each health professional (doctor/GP, midwife and PHN), women were also asked if they were able to talk about things that were troubling them concerning their own health and wellbeing, with a list of nine statements they could endorse. Examples of statements include: ‘Yes, my ‘health professional’ makes it easy for me to talk about anything that is concerning to me’; ‘No, I go to see the ‘health professional’ about my baby, not myself’; and ‘I don’t talk to my ‘health professional’ because I am worried he/she will think I am not coping’. These statements/questions were shared by investigators on the Australian Maternal Health Study (Brown et al., 2006). The proportion of women endorsing each statement is reported at three months postpartum, and combined into a composite variable across 6, 9 and 12 months.

Women were also asked whether or not their healthcare professional had asked them directly about a list of eight common postpartum health issues e.g., leakage or involuntary loss of urine. We report on two health issues that are most relevant to this paper – ‘tiredness/exhaustion’ and ‘feeling depressed or low’. Tiredness/exhaustion has been highlighted as very common for mothers in the postpartum period, but even more common for women reporting mental health issues (Giallo et al., 2016; Hannon et al., 2023).

#### Qualitative data

At each postpartum time point, women were invited to respond to the open text question “Looking back over the time in the past three months, would you like to have had more emotional support?”

## Data analysis

### Quantitative

Proportions are reported as a fraction of the women who responded to each item at each time point. Univariable logistic regression was used to examine the frequency of visits for those who reported anxiety/depressive symptoms compared to women who did not at each time point. Univariable logistic regression was used to examine being able to talk with their health professional at three months and 6–12 months postpartum for women reporting anxiety/depressive symptoms compared to those who did not at each of these time points.

### Qualitative

A total of 1,412 comments were made across the four postpartum surveys. Using manifest content analysis, data were extracted from comments that were relevant to (i) interactions with, and perceptions of, healthcare professionals, (ii) factors that facilitate or inhibit discussion of mental health/sensitive issues with healthcare professionals and (iii) factors that facilitate or inhibit accessing mental health care. Manifest content analysis is a structured analysis and description of observable components of textual data (Graneheim & Lundman, 2004; Kleinheksel et al., 2020), it identifies and reports the direct and explicit content of qualitative data, without the creation of themes or subjective interpretation. The manifest content analysis was conducted by a single coder,

## Results

### Sample

The baseline survey was completed by 3009 eligible women (122 excluded due to being multiparous, having miscarriage, stillbirth, or baby admitted to NICU). For this paper, the 437 women who only completed the antenatal survey were excluded, leaving data from 2572 women as the sample.

Socio-demographic characteristics of the sample are reported in Table 1. Almost half the women were 30–34 years of age at enrolment in pregnancy with their first child (44.8%), with the majority born in Ireland (72.6%) or another EU country (20.4%). One in three had postgraduate education (29.2%). The majority of women gave birth at term (37–41.9.9 weeks gestation, 92.2%), with a third each having a spontaneous vaginal birth, operative vaginal birth or caesarean section.

**Table 1:** Socio-demographic background and birth outcomes in the sample (*n* = 2572)

Baseline (pregnancy)	*n*	%
Mothers age		
17–24 years	167	6.5
25–29 years	532	20.7
30–34 years	1149	44.8
35 + years	716	27.9
Country of birth		
Ireland	1845	72.6
EU country	518	20.4
Non-EU country	179	7.0
Relationship status		
Partner	2489	97.1
No partner	74	2.9
Postgraduate Qualification		
Yes	1807	70.8
No	747	29.2
Paid employment		
Yes	2329	90.8
No	237	9.2
Birth outcomes		
Weeks’ gestation at birth		
Preterm (< 36.9 weeks)	150	5.9
Term (37–41.9.9 weeks)	2364	92.2
Post-term (42 + weeks)	49	1.9
Birth weight (hospital record)		
Less than 2500 g	102	4.2
2500–3999 g	2014	82.7
4000 g or more	319	13.1
Mode of birth		
Spontaneous vaginal	848	34.4
Operative vaginal	813	32.9
Caesarean Section	807	32.7
Total	2572	100

### Presentation of results

The following provides an integrated presentation of the quantitative and qualitative findings from the longitudinal data collection under four headings:

i) Anxiety/Depressive symptoms in the first year postpartum
ii) Contacts with healthcare professionals across the first year postpartum
iii) Contacts with healthcare professionals by report of anxiety/depressive symptoms
iv) Talking to healthcare professionals about concerns

i) *Anxiety*/*depressive symptoms in the first year postpartum*

**Fig. 1:**
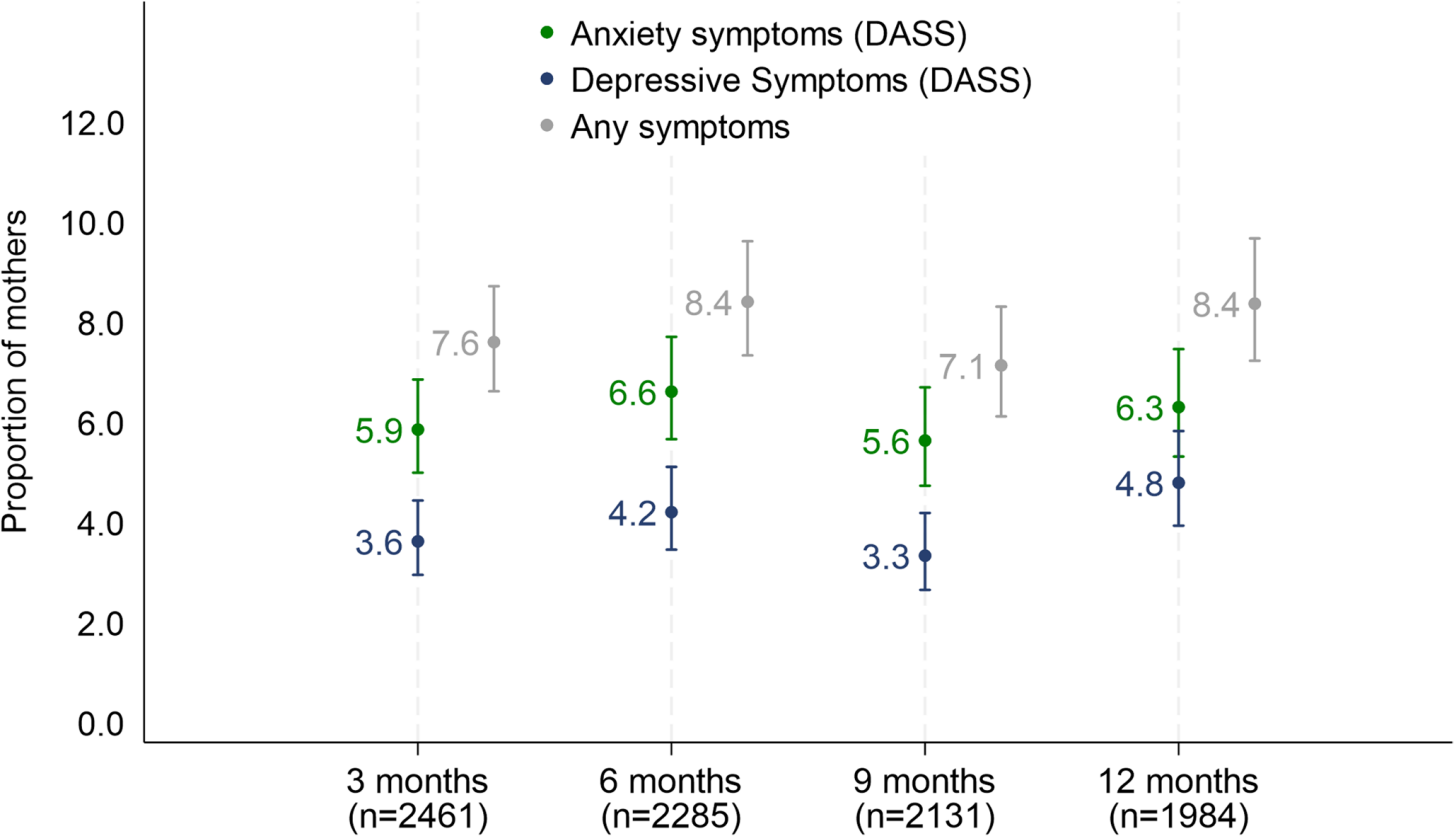
Proportion of mother reporting anxiety and depressive symptoms in the first year postpartum

Between 5.6% and 6.6% of women reported moderate-severe symptoms of depression alone at any postpartum time-point. Between 3.3% and 4.8% of women reported moderate-severe symptoms of anxiety alone at any time-point. (see Fig. 1). Between 7.1 and 8.4% of women reported anxiety/depressive symptoms at each of the time points (three, six, nine, and 12 months postpartum).

ii) *Contacts with healthcare professionals across the first year postpartum*

**Fig. 2:**
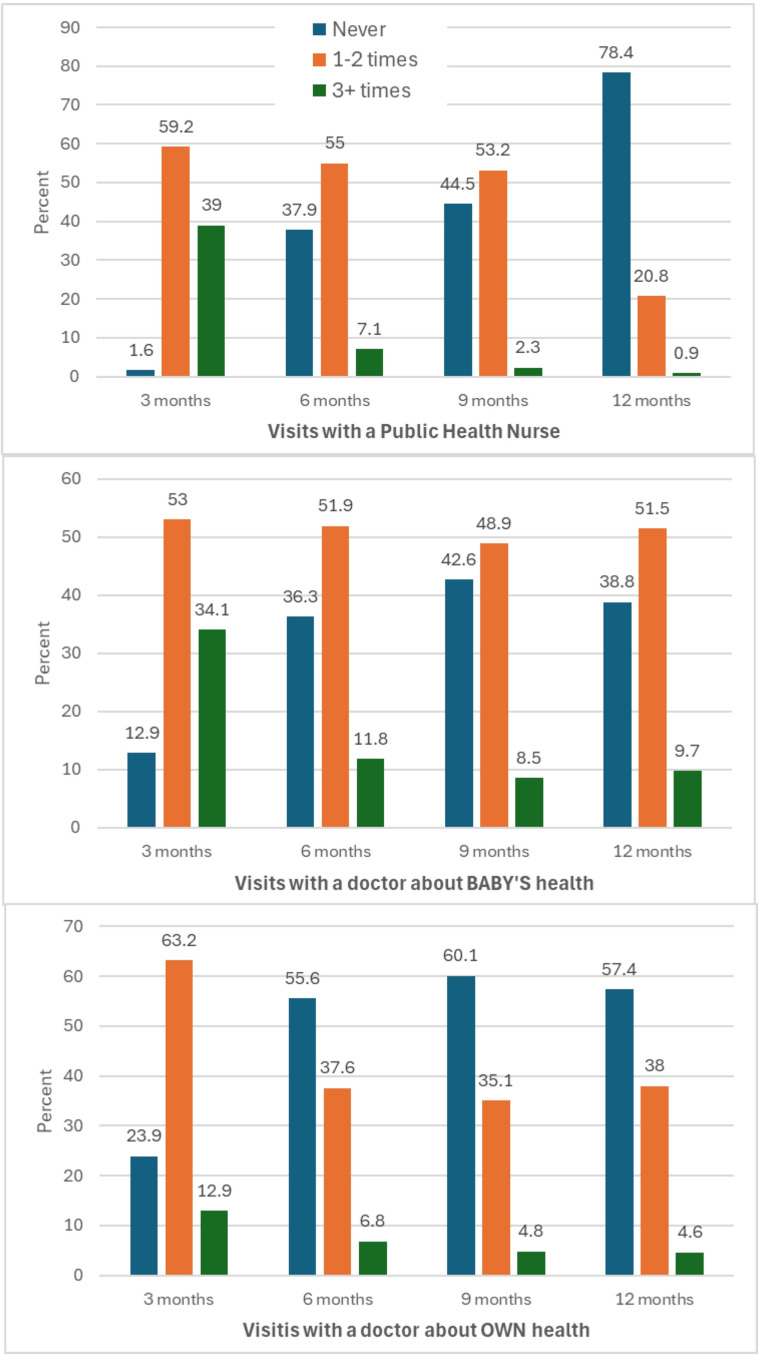
Proportion of mothers (who report depressive and/or anxiety symptoms) visiting each type of health professional at each time point in the first year postpartum Health care across the first year postpartum and experiences of women with anxiety/depressive symptoms: a longitudinal cohort of first-time mothers in Ireland (MAMMI)

Contacts with healthcare professionals at three, six, nine, and 12 months postpartum are described in Table 2 and visually presented in Fig. 2.

**Table 2:** Report of depressive and/or anxiety symptoms and visits with health professionals in the first year postpartum (*n* = 2461)

	3 months	6 months	9 months	12 months
	*n* = 2461	*n* = 2285	*n* = 2131	*n* = 1984
Saw midwife				
Never	1700 (69.3)			
1–2 times	284 (11.6)			
3 or more	469 (19.1)			
Saw public health nurse				
Never	39 (1.6)	860 (37.9)	939 (44.5)	1547 (78.4)
1–2 times	1459 (59.2)	1249 (55.0)	1123 (53.2)	410 (20.8)
3 or more	966 (39.2)	161 (7.1)	49 (2.3)	17 (0.9)
*YES PHN asked about tiredness/exhaustion*	1707 (71.1)^2^	638 (45.1)	343 (29.7)	111 (26.1)
*YES PHN asked about feeling depressed or low*	1548 (64.5)	587 (41.5)	266 (23.1)	78 (18.3)
Saw doctor about baby’s health				
Never	311 (12.9)	777 (36.3)	842 (42.6)	716 (38.8)
1–2 times	1276 (53.0)	1112 (51.9)	968 (48.9)	951 (51.5)
3 or more	822 (34.1)	253 (11.8)	168 (8.5)	180 (9.7)
Saw doctor about OWN health				
Never	583 (23.9)	1262 (55.6)	1280 (60.1)	1139 (57.4)
1–2 times	1539 (63.2)	854 (37.6)	748 (35.1)	754 (38.0)
3 or more	314 (12.9)	154 (6.8)	102 (4.8)	91 (4.6)
*YES DR asked about tiredness/exhaustion*	1180 (51.7)	474 (29.6)	300 (22.4)	291 (21.3)
*YES DR asked about feeling depressed or low*	1169 (51.2)	409 (25.6)	238 (17.8)	196 (14.3)

^1^ Percentages calculated with denominator equal to non-missing values

^2^ At three months the question asked was- ’Has your midwife or public health nurse asked you directly about…’

### Three months postpartum

One in three women saw a midwife at least once in the first three months postpartum. Almost all women had visited/or been visited by a PHN (98.4%), with the majority seeing a PHN 1–2 times. The majority had visited a doctor/GP about their baby’s health (87.1%) and their own health (76.1%). In the first three months postpartum, the majority of women were directly asked by their midwife and/or PHN about tiredness/exhaustion and feeling depressed or low. Half the women reported that their doctor/GP also directly asked them about these issues (51.7% and 51.2% respectively).

Women’s comments regarding emotional support from healthcare professionals at three months postpartum were diverse; most reported satisfaction with the support they received from the GPs, PHNs and other healthcare professionals who actively asked about their mental wellbeing:

*Had really good support from family and friends and GP who were aware of my history with depression*,* also district nurse was very helpful.* (3 months postpartum)

*My GP and PHN have always asked me whether I’m feeling ok.* (3 months postpartum)

Some women’s comments suggested that their care felt limited or was withdrawn too soon in the postpartum period:

*I had weekly support with antenatal classes and check-ups*,* building up a relationship with my midwife. From the week prior to birth I haven’t seen her. All support just suddenly disappeared when I needed it most.* (3 months postpartum)

*I feel this support is not there*,* public health nurse only calls to house a week after baby born- hasn’t hit you yet what’s happening!* (3 months postpartum)

Others indicated that, although the care they received was helpful, emotional and physical health checks specific for mothers’ wellbeing could be a beneficial addition to postpartum maternal care:

*For a first time Mum and even past the 6 weeks check is very scary*,* exciting*,* and all new and brings lots of emotions on top of the tiredness and hormones. It would have been nice to have someone check in with me besides the six week check.* (3 months postpartum)

*Be good to talk to someone just about you after birth*,* even if feeling good*,* still be good to talk about how life changes with baby*,* Health nurse does help though.* (3 months postpartum)

*Lots of people ask ' how are you’ and when you are generally well it’s easy to just brush off the question. Nobody*,* not even health care workers asks specifics (for example) headaches? how’s your back? Unless something is very wrong at time of question other small things are hard to discuss and over time they build up.* (3 months postpartum)

There was some frustration that limited and baby-focused postpartum care meant that women had to initiate help seeking:

*I have felt a complete lack of professional emotional support. If you want professional help you have to go to your GP and seek it out. The district nurses are interested in the babies mainly but mums need support*,* and dads. It is a big happy change.* (3 months postpartum)

There were multiple comments suggesting that having contact with someone who functioned as an impartial emotional support in the postpartum period would benefit some women’s wellbeing:

*I have very supportive friends and family but maybe an outside support would be beneficial*,* e.g. another midwife visit for the Mum before 3 months for a chat on how she’s feeling as the 3 months visit was beneficial to me but a bit too long to wait for.* (3 months postpartum)

*Someone besides friends*,* a healthcare worker or counsellor would have really helped.* (3 months postpartum)

### Six months postpartum

At six months postpartum, half the women reported seeing a PHN and a doctor/GP (about their baby’s health), and a third had seen a doctor/GP about their own health 1–2 times in the past three months (i.e., between 3 and 6 months postpartum). Notably, one in three women had NOT seen a PHN or doctor/GP (about their baby’s health), and just over half had not seen a doctor/GP about their own health (55.6%). Of the women who had seen a PHN, less than half had been directly asked about tiredness/exhaustion (45.1%) or feeling depressed or low (41.5%), and even fewer had been asked by their doctor/GP (29.6% and 25.6% respectively).

### Nine months postpartum

At nine months postpartum, contacts with health professionals were similar to six months, albeit slightly less frequent. A lower proportion of the women were directly asked about tiredness/exhaustion or feeling depressed or low by their PHN (29.7% and 23.1% respectively) or doctor/GP (22.4 and 17.8% respectively).

### Twelve months postpartum

At 12 months postpartum, the majority of women had not seen a PHN (78.4%) or doctor/GP (about their own health, 57.4%) in the previous three months. Just over half of the women reported seeing a doctor/GP about their baby’s health 1–2 times (51.5%). Enquiry about tiredness/exhaustion or feeling depressed or low remained low for both PHNs and doctors/GPs.

Decreased contact with healthcare professionals, and infrequent enquiries regarding mothers’ health and wellbeing from healthcare professionals was a persistent theme in the comments made in the 6–12 months postpartum surveys:

*Having someone who asks direct questions is very useful*,* but it’s rarely the case*,* conversations tend to be general & fleeting.* (9 months postpartum)

*Six week check-up is terrible as I felt forced to combine mine and my baby’s GP visit*,* and I am a new Mam*,* 100 questions about baby and little time for me.* (6 months postpartum)

*Again I think professional review/service in this area is pivotal to mental health. There is so much contact during pregnancy then nothing (almost) post-natal.* (12 months postpartum)

iii) *Contacts with health professionals by report of anxiety/depressive symptoms*

Table 3 reports the frequency of contacts with healthcare professionals and the proportion of women reporting anxiety/depressive symptoms for each time point. Logistic regression was used to examine if there was a difference in the report of anxiety/depressive symptoms by number of visits. The most common category for women was 1–2 visits to a PHN or a doctor/GP about their baby’s health at most time points, so this was set as the reference category.

**Table 3:** Proportion of women reporting anxiety/depressive symptoms for each level of visits to health professionals over the first year postpartum

	3 months		6 months		9 months		12 months	
	*n* = 2429		*n* = 2257		*n* = 2117		*n* = 1965	
	n (%)	OR^1^ [95%CI]	n (%)	OR [95%CI]	n (%)	OR [95%CI]	n (%)	OR [95%CI]
Midwife								
Never	132 (7.8)	0.8 [0.5–1.3]						
1–2 times	26 (9.2)	1.0 [ref]						
3 or more	28 (6.0)	0.6 [0.4–1.1]						
PHN								
Never	4 (10.3)	1.4 [0.5–4.1]	73 (8.5)	1.1 [0.8–1.5]	72 (7.7)	1.1 [0.8–1.5]	125 (8.1)	0.8 [0.6–1.2]
1–2 times	107 (7.3)	1.0 [ref]	100 (8.0)	1.0 [ref]	79 (7.1)	1.0 [ref]	39 (9.7)	1.0 [ref]
3 or more	76 (7.9)	1.1 [0.8–1.5]	19 (11.9)	1.6 [0.9–2.6]	1 (2.1)	nc^2^	2 (11.8)	nc
GP Baby’s health								
Never	15 (4.8)	0.7 [0.4–1.2]	40 (5.2)	0.6 [0.4–0.8]	46 (5.5)	0.7 [0.5 −1.0]	47 (6.6)	0.7 [0.5 −1.0]
1–2 times	88 (6.9)	1.0 [ref]	99 (8.9)	1.0 [ref]	76 (7.9)	1.0 [ref]	87 (9.2)	1.0 [ref]
3 or more	81 (9.9)	1.5 [1.1 −2.0]	39 (15.6)	1.9 [1.3–2.8]	20 (11.9)	1.6 [0.9–2.7]	16 (9.0)	1.0 [0.6–1.7]
GP OWN health								
Never	29 (5.0)	0.6 [0.4–0.9]	62 (4.9)	0.5 [0.3–0.7]	61 (4.8)	0.6 [0.4–0.8]	65 (5.8)	0.5 [0.4–0.8]
1–2 times	124 (8.1)	1.0 [ref]	85 (10.0)	1.0 [ref]	61 (8.2)	1.0 [ref]	75 (10.0)	1.0 [ref]
3 or more	32 (10.2)	1.3 [0.9 −2.0]	44 (28.6)	3.6 [2.4–5.4]	29 (29.0)	4.6 [2.7–7.5]	24 (26.7)	3.3 [1.9–5.5]

^1^ Odds of reporting depressive/anxiety symptoms by number of visits e.g. Women who visited a GP 3 or more times for their baby’s health had one and half times the odds (1.5) of depressive/anxiety symptoms compared to women visiting their GP 1–2 times for their baby’s health

2 nc=”Not calculated” due to small numbers

The proportion of women reporting anxiety/depressive symptoms was no different by the number of visits for either the midwives or PHNs (see Table 3). For example, a similar proportion of women who ‘never’ saw a midwife reported anxiety/depressive symptoms (7.8%, OR = 0.8, 95%CI 0.5–1.3) as did women who saw the midwife 1–2 times (9.2%) or 3 or more times (6.0%, OR = 0.6 95%CI 0.4–1.1). However, women with anxiety/depressive symptoms had a higher number of visits to the doctor/GP for their own and their baby’s health. For example, at three months postpartum, 9.9% of the women who visited a doctor/GP three or more times for their baby’s health reported anxiety/depressive symptoms compared to 6.9% of those visiting 1–2 times i.e., women seeing the doctor/GP three or more times had one and a half times the odds of anxiety/depressive symptoms at three months (OR = 1.5, 95%CI 1.1 −2.0) and at six months postpartum (OR = 1.9, 1.3–2.8) compared to women seeing the doctor/GP 1–2 times. The women who ‘never’ saw a doctor/GP about their baby’s health had lower odds of reporting anxiety/depressive symptoms at six, nine and 12 months postpartum.

Similar findings were observed for visits to the doctor/GP about their own health. Compared to women visiting the doctor/GP 1–2 times, women with anxiety/depressive symptoms had lower odds of ‘never’ visiting the doctor/GP and higher odds of visiting more often (3 or more times). This was observed at three, six, nine and twelve months postpartum. For example, at 12 months postpartum, women visiting a doctor/GP three or more times for their own health had three times the odds of anxiety/depressive symptoms compared to women visiting 1–2 times (OR = 3.3, 95%CI 1.9–5.5).

Women’s comments indicated that there were material barriers to obtaining support from healthcare professionals in the postpartum period including costs, limited time to attend appointments while balancing workload/childcare responsibilities, and long wait times between seeking care and receiving appointments.

*I feel that after you give birth there is no real support from health care unless you pay.* (3 months postpartum)

*Initially I contacted [counselling support service] therapy. It was 75 Euro so I couldn’t afford it. 55 Euro + 20 Euro registration*. (6 months postpartum)

*I have started seeing a counsellor*,* service was offered to me after I had the baby because of the birth. I accepted the offer then*,* but only got called for appointment now. If I was just offered now I don’t think I would have felt I needed it*. (9 months postpartum)

*I would have liked to go back to my counsellor but I couldn’t because of baby/husband working*. (9 months postpartum)

*Counselling is expensive & babies not allowed attend.* (12 months postpartum)

*Counsellor waiting list was months long and once a week is not enough.* (12 months postpartum)

*Suppose not having close family around and not being able to afford childcare contributes.* (12 months postpartum)

Comments showed that some women thought that mental health problems ‘should’ resolve early in the postpartum and that this was a stigma-based barrier to accessing healthcare in the longer term:

*Feel a bit silly to still be struggling at this stage.* (6 months postpartum)

*A bit embarrassed to admit.* (6 months postpartum)

*Doesn’t seem to be the ‘done thing’ feel like you might let yourself down if you complain or seem ungrateful.* (9 months postpartum)

However, there were also indications that contact with healthcare professionals led to receiving care and recovery.

*Was GP visit which led to counselling. People feel you ‘should’ be fine by now!* (12 months postpartum)

*I have postnatal depression*,* but an amazing husband and doctor*,* and they have helped me through (along with medication) and I’m feeling much better. I am always asked how I am feeling*. (12 months postpartum).

iv) *Talking to health professionals about concerns*

Women were asked if they “felt able to talk to your PHN/doctor about things that are troubling you concerning your own health and wellbeing?” and could then endorse a list of nine options (see Table 4). There were few differences in women’s responses to the statements at six, nine and 12 months postpartum, and accordingly these were combined for clarity of presentation.

**Table 4:** Perceptions of being ‘able to talk about things that are troubling you concerning your health and wellbeing” for women who reported depressive and/or anxiety symptoms

	3 months postpartum			6–12 months postpartum			
	All women *n* = 2429	Women with depressive/anxiety symptoms		All women *n* = 1891	Women with depressive/anxiety symptoms		
	n (%)	n (%)	OR [95%CI]	n (%)	n (%)		OR [95%CI]
Talking to public health nurse							
Yes, they make it easy talk to about anything concerning me	1472 (62.1)	75 (41.4)	0.4^***^ [0.3–0.5]	1229 (65.2)	132 (47.3)		0.4^***^ [0.3–0.5]
Yes, I can talk and they are very supportive and reassuring	580 (24.5)	37 (20.4)	0.8 [0.5–1.1]	656 (34.9)	75 (27.0)		0.6^**^ [0.5–0.9]
No, I go to see them about the baby not myself	394 (16.7)	48 (26.5)	1.9^***^ [1.4–2.7]	609 (32.4)	123 (44.2)		1.8^***^ [1.4–2.4]
I can talk about some issues but others I am not comfortable talking about	213 (9.0)	23 (12.7)	1.5 [1.0–2.4.0.4]	273 (14.5)	68 (24.5)		2.2^***^ [1.6–3.0.6.0]
Yes, but they are often busy and don’t seem to have the time	138 (5.8)	15 (8.3)	1.5 [0.9–2.7]	164 (8.7)	34 (12.2)		1.6^*^ [1.1–2.4]
No point talking about my health because they can’t fix any of my problems	64 (2.7)	16 (8.8)	4.3^***^ [2.4–7.8]	82 (4.4)	28 (10.1)		3.2^***^ [2.0–5.2.0.2]
I don’t talk to them because I am worried they will think I am not coping	59 (2.5)	19 (10.5)	6.3^***^ [3.6–11.1]	68 (3.6)	33 (11.9)		6.0^***^ [3.7–9.9]
I don’t talk to them because I am concerned they will want me to do something that might make the situation worse	18 (0.8)	6 (3.3)	nc^2^	24 (1.3)	12 (4.3)		6.0^***^ [2.7–13.4]
There are some issues I don’t talk about because they might tell someone else	14 (0.6)	5 (2.8)	nc	23 (1.2)	14 (5.0)		9.4^***^ [4.0–21.9.0.9]
Talking to a doctor/GP							
Yes, they make it easy talk to about anything concerning me	1494 (66.0)	84 (47.2)	0.4^***^ [0.3–0.6]	1648 (77.4)	238 (71.7)		0.7^**^ [0.5–0.9]
Yes, I can talk and they are very supportive and reassuring	642 (28.4)	39 (21.9)	0.7^*^ [0.5–1.0.5.0]	968 (45.4)	153 (46.1)		1.0 [0.8–1.3]
No, I go to see them about the baby not myself	147 (6.5)	23 (12.9)	2.3^***^ [1.5–3.8]	359 (16.9)	66 (19.9)		1.3 [0.9–1.7]
I can talk about some issues but others I am not comfortable talking about	288 (12.7)	48 (27.0)	2.8^***^ [2.0–4.1.0.1]	396 (18.6)	103 (31.0)		2.3^***^ [1.8–3.0.8.0]
Yes, but they are often busy and doesn’t seem to have the time	280 (12.4)	27 (15.2)	1.3 [0.8–2.0.8.0]	313 (14.7)	67 (20.2)		1.6^**^ [1.2–2.2]
No point talking about my health because they can’t fix any of my problems	43 (1.9)	11 (6.2)	4.2^***^ [2.1–8.5]	67 (3.1)	31 (9.3)		5.0^***^ [3.1–8.3]
I don’t talk to them because I am worried they will think I am not coping	52 (2.3)	20 (11.2)	8.1^***^ [4.5–14.5]	57 (2.7)	30 (9.0)	6.5^***^ [3.8–11.1]	
I don’t talk to them because I am concerned they will want me to do something that might make the situation worse	8 (0.4)	3 (1.7)	nc	15 (0.7)	6 (1.8)	nc	
There are some issues I don’t talk about because they might tell someone else	6 (0.3)	3 (1.7)	nc	20 (0.9)	9 (2.7)	nc	

^1^ Odds ratio = odds that women with depressive and/or anxiety symptoms respond yes to each item compared to women not reporting symptoms

2 nc= “Not Calculated” due to small numbers- (^*^ *p* < 0.05, ^**^ *p* < 0.01, ^***^ *p* < 0.001)

Supplementary Table 1. Report of depressive and/or anxiety symptoms and visits with health professionals in the first year postpartum for the subsample of women who completed all time points (*n* = 1839)

#### Public health nurse (PHN)

At three months postpartum, the majority of women felt that their PHN made it easy for them to talk about their concerns (62%). However, only one in four women reported that their PHN was supportive and reassuring (24.5%), and almost one in five reported that the PHN visit was about their baby rather than themselves (16.7%). Almost one in ten women felt that there were some issues they were comfortable discussing with their PHN but others they were not comfortable talking about (9%). Across the six to 12 months postpartum period, the majority of women again reported that the PHN made it easy to talk about any concerns (65.2%), and a slightly higher proportion found their PHN reassuring and supportive (34.9%) than at three months. However, a higher proportion also reported that the PHN was there for the baby rather than themselves (32.4%), and felt that they could talk about some issues but not others (14.5%). Less than 10% of the women endorsed items describing reasons why women might *not* talk to their PHN (See Table 4).

There were significant differences in how women with anxiety/depressive symptoms responded to these items, compared to women without symptoms. One in four women with anxiety/depressive symptoms agreed that contacts with the PHN were about their baby’s health rather than their own (26.5%). This represented almost twice the odds of agreeing with this statement compared to women not reporting symptoms (OR = 1.9, 95%CI 1.4–2.7). They were also one and a half times as likely to report that they were comfortable talking about some issues but not others (OR = 1.5, 95%CI 1.0–2.4.0.4). Compared to women not reporting symptoms, women with anxiety/depressive symptoms had 4–7 times the odds of endorsing the items describing reasons why women did not talk to their PHN including that there was no point talking to their PHN because they could not help with their problems; they do not talk because they were worried the PHN would think they are not coping; might want them to do something that might make the situation worse; or might tell someone else. Wide confidence intervals were observed as the number of women endorsing these items remained small.

The findings were almost identical for the 6–12-month postpartum period, with women who were experiencing anxiety/depressive symptoms significantly less positive about their capacity to talk about things that were troubling them, compared to women not reporting symptoms.

#### Doctors/GPs

At three months postpartum, a majority of women reported that their doctor/GP made it easy to talk about things that concerned them, however only one in four reported that they found the doctor/GP supportive and reassuring (28.4%). One in ten women reported that the doctor/GP was often busy and did not seem to have the time (12.4%) and that they felt comfortable talking about some issues but not others (12.7%). As with the PHNs, less than 10% of women endorsed items describing reasons why they might *not* talk to their doctor/GP such as being worried the doctor/GP would think they were not coping (2.3%).

Again, there were differences in how women with anxiety/depressive symptoms responded to these items. Less than half the women with anxiety/depressive symptoms reported that their doctor/GP made it easy to talk about things that were concerning them (47%, OR = 0.4, 95%CI 0.3–0.6). Compared to women without symptoms, women with anxiety/depressive symptoms were more than twice as likely to agree that doctor/GP visits were about the baby rather than their own health (OR = 2.3, 95%CI 1.5–3.8), and had nearly three times the odds of agreeing that they were comfortable talking about some issues but not others (OR = 2.8, 96%CI 2.0–4.1). One in ten women with anxiety/depressive symptoms agreed with the statement “I don’t talk to them because I am worried they will think I am not coping” (11.2%). Women with anxiety/depressive symptoms had 4–12 times the odds of agreeing with items describing reasons why they might not talk to their doctor/GP, compared to women not reporting symptoms (see Table 4).

Very similar findings were observed at six-to-12-months. Most women reported that the doctor/GP made it easy to talk about things concerning them (77.4%); however, less than half felt that the doctor/GP was very supportive and reassuring (45.4%). Almost one in five women reported they were comfortable talking about some things with the doctor/GP and not others (18.6%) and more than one in ten reported the doctor/GP was often busy and did not seem to have the time (14.7%).

Women with anxiety/depressive symptoms were again significantly less positive about their contacts with doctors/GPs compared to women not reporting anxiety/depressive symptoms (See Table 4). Women with symptoms had lower odds of agreeing that the doctor/GP made it easy to talk about their concerns (OR = 0.7, 95%CI 0.5–0.9) and had twice the odds of agreeing that they were comfortable talking about some issues but not others and that their doctor/GP was often busy and did not seem to have the time (see Table 4). Again, women with symptoms had very high odds of endorsing items describing reasons why they might *not* talk to their doctor/GP compared to women not reporting symptoms, but again the estimates had wide confidence intervals as the numbers endorsing each item remained small (see Table 4).

Comments relating to women’s confidence and comfort in speaking to healthcare professionals about mental health or sensitive issues varied. A majority of comments were positive and referenced positive support and easy interactions with healthcare professionals.

*GP*,* public health nurse and nurse at GP surgery were very compassionate and helpful.* (3 months postpartum)

*Have spoken to GP in past week and feel much better*,* so support really helps outside of the family*. (6 months postpartum)

However, a number of women wrote about attempts to discuss their concerns where they felt dismissed or not listened to;

*I went to see the support midwife and was given the name of a book to get; I didn’t feel like my problems were fully listened to and proper support provided.* (3 months postpartum)

*I did try and chat to my GP*,* but he just spoke for me and I didn’t see the point in continuing.* (6 months postpartum)

*Sometimes I feel as though I am being brushed off.* (6 months postpartum)

Other comments described fears of what may happen if they did disclose concerns, often centred on fears of being judged as ‘not coping’ as a mother, of being labelled mentally ill, or being medicated.

*Despite wanting emotional support*,* I find it difficult to admit that I need it. Don’t want anyone thinking I can’t cope.* (3 months postpartum)

*Everyone has been great but I’m afraid to say too much in case they try to label it PND and medicate me. I don’t want to be a zombie or falsely happy.* (3 months postpartum)

*Feel stressed mainly due to baby crying/feel that as a mother I should be able to console/calm baby. Don’t want others to judge if I say I’m having trouble with baby.* (3 months postpartum)

*Some people look at you*,* almost like you’re not coping if you try talking about those things.* (12 months postpartum)

Of note were the frequent comments on the need for additional care pathways that extend beyond three months postpartum and that provide an opportunity for greater emotional support for women across the full first year of motherhood. Women indicated that having access to a healthcare professional, dedicated maternal/baby health care spaces, more frequent appointments and/or call lines would be beneficial to women in the postpartum period.

*I feel there should be far greater and regular/frequent support from health professionals for (new) mothers and fathers.* (6 months postpartum)

*Be good to have a phone line or online support.* (6 months postpartum)

*I am receiving help with my wellbeing*,* but I think a nurse or carer one-on-one*,* drop in centre and bring baby would be more beneficial to me as sometimes I feel like a number in the hospital system. I feel vulnerable at times and I don’t like to burden my partner and family with how I am feeling.* (12 months postpartum)

*I think all mothers should be checked with via phone*,* text*,* or face to face weekly to ensure they’re coping!!! (Health visitor or nurse)* (12 months postpartum).

*Support from outside the family or friends would be great*,* just someone checking in who doesn’t need to be protected or non-judgemental*. (12 months postpartum)

## Discussion

The postpartum period and the transition to motherhood represent a time of vulnerability to the recurrence or development of mental (Falah-Hassani et al., 2017) and physical health problems (Daly et al., 2022; Gartland et al., 2016). Medical and child development appointments in the postpartum period are opportunities for healthcare professionals to check in with mothers about their physical and mental wellbeing and provide appropriate care or referral.

Most women in the current study indicated that they had at least one contact with a healthcare professional, for either their own or their baby’s health in the first three months postpartum. However, the results of this study also indicated that, although provided as a standard and free part of postpartum maternity care, a significant percentage of women did not receive or did not attend a postpartum health check with a GP for themselves (*n* = 583, 23.9%) or their baby (*n* = 311, 12.95%) in the first three months postpartum. The findings from this study are similar to those of the National Maternity Experience Survey (National Care Experience Programme, 2020), in which 1% of women reported that they had not been visited at home by a PHN after birth, and 15% said that they had not received the 2-week check-up from their doctor/GP for their baby’s health (National Care Experience Programme, 2020). Comparably, the current research shows that 1.6% did not receive a PHN visit and 12.9% did not receive a GP visit. The qualitative data in this study illustrated that women are concerned about the decreasing contact that they have with healthcare professionals and not being asked often enough about their health in the first year postpartum. These findings align with previous qualitative research showing that women view the conclusion of maternity care at 6 weeks postpartum as an arbitrary and premature cut-off, and that they require, and would benefit from, longer term emotional and information support from trusted professionals and resources (Hannon, Newnham, et al., 2022).

Previous research with PHNs in Ireland found that 87% of PHN's self-reported that they asked women about mental health as part of their regular perinatal clinical practice (Higgins et al., 2018). Women in the current study, however, reported lower frequency of enquires from HCPs; 64.5% of women reported that a PHN asked them about feeling depressed or low at three months postpartum, and 71.1% reported being asked about tiredness and exhaustion. A lower proportion again reported that their GP asked them about feeling depressed or low (51.2%) or about tiredness and exhaustion (51.7%) at three months postpartum. Additionally, three months postpartum had the highest proportion of doctors/GPs or PHNs asking, with reports significantly decreasing at each subsequent time point. In the Irish National Maternity Experience Survey, 29% of women felt that a practice nurse/midwife or GP had not spent enough time talking to them about their mental health at their postpartum appointment (National Care Experience Programme, 2020). These investigations, taken together, indicate some discrepancy between professionals’ accounts of their clinical practice and women’s reports of being asked about mental health. Mothers’ qualitative comments within the current research may offer some insight as to reasons for this discrepancy; *how* mothers are asked about their physical and mental health is important. Some mothers felt that they were not asked direct questions about issues and that the conversation was too general or infant focused.

The current research offers women’s perspectives on their experiences and cannot speak to the factors that may underlie enquiry or non-enquiry from a healthcare professional’s perspective. However, international and Irish research suggests several barriers to healthcare professionals asking women about their perinatal mental health. For example, midwives describe having too many tasks to complete during brief appointments, a lack of knowledge around perinatal mental health and a need for specific training (Williams et al., 2016). GPs mention hesitation in opening conversations about mental health where disclosures may lead to longer consultations and impact on the time available for other patients. A lack of skill and confidence in initiating mental health conversations and limited knowledge of perinatal mental health problems have also been noted as a potential barrier for PHNs (Higgins et al., 2018). Additionally, awareness of the demand for mental health services, long waiting lists and the difficulties involved in accessing appropriate care may leave HCPs hesitant to raise questions around mental health, particularly if they feel that they cannot offer adequate and timely support following a disclosure (Health Service Executive, 2006). Consequently, the subject of insufficiently-resourced mental health services is a salient and documented consideration for policy makers who may affect change and improvement (European Commission, June 2023).The current findings also illustrate that a higher number of visits to a GP in the postpartum period was more likely for women reporting anxiety/depressive symptoms. Interestingly, qualitative research with GPs in Ireland found that some believe that maternal anxiety may be identified by, or is associated with, a higher number of child consultations (Noonan, Doody, et al., 2018). This was supported in the current study only at three and six months postpartum. There was a much stronger association between anxiety/depressive symptoms and the number of visits to a GP for a woman’s *own health* at six (OR = 3.6, 95%CI 2.4–5.4), nine (OR = 4.6, 95%CI2.7 −7.5), and 12 (OR = 3.3, 95%CI 1.9–5.5) months postpartum. This is good news if these visits are taken as opportunities for healthcare professionals to initiate and follow up mental health conversations and connect women with appropriate care pathways.

However, caution should be exercised in the interpretation of these results. These findings demonstrate a correlational, not causal, association between anxiety/depressive symptoms and increased frequency of GP visits. Therefore bidirectional influences should be considered; for example, research demonstrates that women experience high levels of health problems in the first year postpartum and beyond; including depression, exhaustion, severe pain, wound and urinary tract infections, viral illnesses, haemorrhoids and constipation, sexual health problems, and urinary incontinence, which may necessitate increased visits to a healthcare professional (Bergström et al., 2017; Gartland et al., 2016; Gmelig Meyling et al., 2023; Hannon et al., 2023; O’Malley et al., 2018). Additionally, experiencing physical illness (Bondesson et al., 2024), or having an ill child (Warmerdam et al., 2020) negatively impacts one mental wellbeing. Therefore, healthcare professionals also need to remain mindful as to the considerable health burden that mothers experience and be careful not to assume that mothers’ concerns about their own or their child’s health are anxiety driven.

Increased odds of reporting anxiety/depressive symptoms by number of visits to a PHN or GP highlight a potential area for improvement in healthcare provision. Ireland currently provides free GP visits for all children up to six years old. However, beyond the six-week check women must cover the costs for their own health appointments unless eligible for a means-assessed GP or general medical card. Figures specific to perinatal women are not available, but 30.8% of the Irish population were medical card holders in 2021 (Department of Health, 2022). Additionally, while free counselling services are offered to medical card holders for a maximum of eight sessions (Health Service Executive, 2022), other mental health treatment must be self-financed. The costs associated with seeking private mental health care were a prohibitive factor noted in the qualitative data at each postpartum time point. Inequitable access to mental health services in Ireland has been noted previously (Cullinan et al., 2016); however, this research shows that mothers face an unseen double fee when seeking treatment, as time taken to attend therapy must also factor in costs for childcare services, and time away from work or family. Private and public counselling services in Ireland may consider these additional challenges for mothers and seek to provide women with flexibility in appointment options, as per women’s comments, this may include offering virtual appointments, and baby welcome sessions.

Mothers giving birth in Ireland may benefit from the development of new, integrated, and extended pathways of accessing physical and mental postpartum healthcare. Extending postpartum care beyond six weeks postpartum, to include contact with a wider range of healthcare professionals, in accessible community-based settings may provide an access point to physical, psychological and social treatments or resources, through which mothers’, and thus families’, overall well-being may be improved. Furthermore, an innovative, multi-disciplinary healthcare service approach pragmatically delivers on a multi-systemic understanding of health and well-being (Ungar, 2021). Policy intervention determines service provision, and policy intervention that enables provision of community resources and free of charge healthcare service availability has the potential to improve women’s physical, psycho-social, and economic well-being. Additionally, new and alternative pathways to accessing postpartum care may also serve to lessen workload issues that remain a challenge in general practice, thereby improving healthcare systems’ functionality. New, integrated, and extended pathways of accessing postpartum healthcare, of course, must be underwritten by policy change, and receive sufficient funding to bring such services to fruition. These suggestions, underpinned by the qualitative and quantitative data of the current study, may provide the next iteration of Ireland’s National Maternity strategy with an avenue for service improvement.

The majority of women reported positive perceptions of PHNs and GPs in the first year postpartum. A total of 62.1% and 65.2% of all women, at 3 and 6–12 months postpartum respectively, indicated that their PHN made it easy for them to talk about concerns. Similarly high percentages were observed for GPs (66.0%, 77.4%). However, women who reported anxiety/depressive symptoms were less likely to endorse this perception of PHNs or GPs. A meta-synthesis of women’s experiences of seeking help for perinatal psychological distress reported that women were influenced by their perception of the attentiveness and availability of healthcare professionals. For example, women were more likely to discuss mental health issues when they perceived that professionals were interested in their wellbeing and less likely when they perceived that they were too busy to provide support (Button et al., 2017).

Women who reported anxiety/depressive symptoms were also more likely to endorse statements indicating fears of being judged (I am worried that they will think I am not coping) or facing undesirable repercussions (I am concerned they will want me to do something that might make the situation worse; I don’t talk about because they might tell someone else). The qualitative data added to these statements, with women reporting fears that their ability as a mother would be called into question, that they may receive a potentially stigmatising label or feared being medicated. The impact of stigma in relation to perinatal mental health help seeking is noted within the literature (Dolman et al., 2013; Nagle & Farrelly, 2018), and some qualitative research has indicated that women perceive medication as a likely, though unwanted outcome from making disclosures (Hannan, 2016). Previous research shows that women have concerns about medication side effects (Eakley & Lyndon, 2022) and women in this research named specific concerns such as negative impacts on their functionality and worry about experiencing dulled affect. The limited research available suggests that women and the general public have fragmented knowledge and misconceptions of symptoms, causes and treatment options for perinatal mental health problems (Daehn et al., 2022). Improving the public’s health literacy on the benefits and limitations of different treatment options at any stage in the life course, combined with affordable and easy access to referral and treatment must be a goal of all effective health systems.

## Strengths and limitations

This research uses quantitative and qualitative data collected at multiple points in the first postpartum year from a large cohort study to investigate complementary objectives. The advantage of this longitudinal design enables a view of change in symptoms and HCP contact across time.

A key limitation should be mentioned; although the women recruited to the study are broadly representative in terms of nationality and age, the sample includes more women living with a partner, educated to a postgraduate level and in paid employment compared to national statistics for first-time mothers giving birth in Ireland at the time of recruitment (Central Statistics Office, 2016). In other words, the sample contains a greater number of participants with indices of socio-economic advantage than the general population, and this sample shows lower endorsement of anxiety and depression symptoms than the general population. Yet several impediments to accessing timely and affordable healthcare, and barriers to making disclosures were identified within the current sample.

Therefore, given the known associations between mental health symptomology and socio-economic disadvantage (Evans-Lacko et al., 2018; Kirkbride et al., 2024), anxiety/depressive symptoms among a more diverse sample are likely to be higher. Likewise, barriers experienced by women with less socio-economic means to accessing health care services and treatment than the current sample are likely to be more pronounced.

Additionally, the study only recruited women who had sufficient levels of English to complete the surveys. It is likely that the experiences of non-English speaking, migrant, first-time mothers in Ireland are not represented within the results, therefore cultural differences in attitudes towards mental health help-seeking (Chen et al., 2020) may not be captured.

Consequently, the findings, and its implications for service provision and policy change, should also be considered in light of the changing demographic characteristics of the population of women giving birth in Ireland, and therefore must account for the additional barriers and increased mental health burdens experience by women with fewer socio-economic resources.

## Conclusion

The research found that a significant percentage of women did not receive or attend a postpartum health check with a GP for themselves (*n* = 583, 23.9%) or their baby (*n* = 311, 12.95%) in the first three months postpartum, even though this service is provided free to all women giving birth in Ireland. To ensure that all women receive and avail of this vital care, future research may explore if this is due to gaps in care provision (lack of GP access), communication (lack of public knowledge concerning healthcare entitlements), or other factors.

Maternity care services in Ireland conclude at six weeks postpartum at which time women must independently seek out and cover the costs for physical and mental health problems associated with pregnancy, birth or the transition to motherhood, a factor which was noted as prohibitive by some women in this study. As healthcare for children under 6 is free and postnatal care for women after six weeks is not, this sends a clear message to new mothers that their health is not important – a message that needs to change if women are ever to receive the quality and quantity of health care they deserve.

Postpartum health or infant developmental appointments are routinely noted by the international literature as opportunities for HCPs to identify women’s health issues and connect them with suitable services and care. Yet, this study found that in Ireland, only 51.7% of women reported that their GP enquired if they were feeling depressed or low, while 64.5% of women reported that their PHN had asked them about these issues in the first three months postpartum, with reports steadily declining thereafter. These data, considered alongside women’s views that postnatal care is predominately baby-focused, and concludes prematurely, supports the need for the development of additional and extended postnatal care pathways. These findings highlight the limitations of current service provision while providing suggestions which may be implemented in Irish maternity care policy.

## Supplementary Information

Below is the link to the electronic supplementary material.

ESM 1 Supplementary Material (DOCX 7.28 KB)

## Data Availability

None.
