# Supplementary material for: Health care across the first year postpartum and experiences of women with anxiety/depressive symptoms: A longitudinal cohort of first-time mothers in Ireland (MAMMI)
Source: Arch Womens Ment Health. 2026 Feb 5;29(1):34. doi: 10.1007/s00737-025-01670-2 (PMC12876076; doi:10.1007/s00737-025-01670-2)
Supplement: Supplementary file 1 — Supplementary Material (DOCX 7.28 KB) [file 737_2025_1670_MOESM1_ESM.docx]

Supplementary Table 1. Report of depressive and/or anxiety symptoms and visits with health professionals in the first year postpartum for the subsample of women who completed all time points (n = 1839).

|  | **3 months** | **6 months** | **9 months** | **12 months** |
| --- | --- | --- | --- | --- |
|  | n=1833 | n=1835 | n=1833 | n=1826 |
| Depressive symptoms (Moderate-Severe) | 105 (5.7) | 117 (6.4) | 101 (5.5) | 113 (6.2) |
| Anxiety Symptoms (Moderate-Severe) | 62 (3.4) | 69 (3.8) | 56 (3.1) | 82 (4.5) |
| Any symptoms (Depressive and/or Anxiety) | 136 (7.4) | 142 (7.7) | 125 (6.8) | 149 (8.2) |
|  |  |  |  |  |
| **Number of times saw midwife** |  |  |  |  |
| Never | 1295 (70.8) |  |  |  |
| 1-2 times | 192 (10.5) |  |  |  |
| 3 or more | 341 (18.7) |  |  |  |
| **Number of times saw PHN** |  |  |  |  |
| Never | 27 (1.5) | 674 (37.1) | 783 (43.3) | 1423 (78.5) |
| 1-2 times | 1073 (58.5) | 1015 (55.8) | 983 (54.3) | 374 (20.6) |
| 3 or more | 735 (40.1) | 130 (7.1) | 43 (2.4) | 16 (0.9) |
| *YES PHN asked ab tiredness/exhaustion* | 1275 (71.2) | 509 (44.3) | 300 (29.6) | 101 (26.0) |
| *YES PHN asked ab feeling low/dep* | 1149 (64.2) | 473 (41.2) | 235 (23.3) | 68 (17.5) |
| **Number of times saw GP about baby's health** |  |  |  |  |
| Never | 238 (13.2) | 646 (37.2) | 724 (42.6) | 672 (39.6) |
| 1-2 times | 957 (53.2) | 907 (52.3) | 839 (49.4) | 855 (50.4) |
| 3 or more | 604 (33.6) | 182 (10.5) | 136 (8.0) | 171 (10.1) |
| **Number of times saw GP about own health** |  |  |  |  |
| Never | 436 (24.1) | 1030 (56.5) | 1100 (60.2) | 1058 (58.1) |
| 1-2 times | 1149 (63.5) | 683 (37.5) | 642 (35.1) | 685 (37.6) |
| 3 or more | 225 (12.4) | 109 (6.0) | 85 (4.7) | 78 (4.3) |
| *YES GP asked ab tiredness/exhaustion* | 880 (51.8) | 366 (28.7) | 240 (21.0) | 248 (20.0) |
| *YES GP asked ab feeling low/dep* | 866 (51.0) | 309 (24.3) | 187 (16.4) | 172 (13.8) |
